# Biosynthesis and Characterization of Silver Nanoparticles and Simvastatin Association in Titanium Biofilms

**DOI:** 10.3390/ph17121612

**Published:** 2024-11-29

**Authors:** Sindy Magri Roque, Ana Carolina Furian, Marcela Kim Takemoto, Marta Cristina Teixeira Duarte, Rafaela Durrer Parolina, Adriano Luís Roque, Nelson Duran, Janaína de Cássia Orlandi Sardi, Renata Maria Teixeira Duarte, Karina Cogo Muller

**Affiliations:** 1 Laboratório de Farmacologia de Antimicrobianos e Microbiologia, Faculdade de Ciências Farmacêuticas, Universidade Estadual de Campinas (UNICAMP), Campinas 13083-970, Brazil; s152591@dac.unicamp.br (S.M.R.); anafurian@hotmail.com (A.C.F.); 2 Departamento de Biociências, Faculdade de Odontologia de Piracicaba, Universidade Estadual de Campinas (UNICAMP), Piracicaba 13414-903, Brazil; takemotomarcela@gmail.com (M.K.T.); mduarte@cpqba.unicamp.br (M.C.T.D.); rafaelaparolina@gmail.com (R.D.P.); 3 Centro Pluridisciplinar de Pesquisas Químicas, Biológicas e Agrícolas—CPQBA, Universidade Estadual de Campinas (UNICAMP), Paulínia 13148-218, Brazil; duartemt@unicamp.br; 4 Programa de Pós Graduação em Medicina (Cardiologia), Universidade Federal de São Paulo (UNIFESP), São Paulo 04021-001, Brazil; adriano.roque@unifesp.br; 5 Laboratório de Carcinogenese Urogenital e Imunoterapia, Universidade Estadual de Campinas (UNICAMP), Campinas 13083-862, Brazil; nelsonduran1942@gmail.com; 6 Divisão de Pesquisa em Odontologia, Universidade de Guarulhos (UNG), Guarulhos 07023-070, Brazil; janasardi@gmail.com

**Keywords:** simvastatin, anti-infective agents, dental implants, silver nanoparticles

## Abstract

Introduction: Simvastatin is an antilipidemic drug that has already demonstrated antibacterial activities on oral and non-oral microorganisms. Silver nanoparticles also exhibit antimicrobial properties, particularly for coating implant surfaces. In this study, we evaluated the effects of combining simvastatin with silver nanoparticles on the formation and viability of biofilms consolidated on titanium discs. Methods: Silver nanoparticles were first biosynthesized using the fungus *Fusarium oxysporum* and then characterized using Dynamic Light Scattering, X-ray Diffraction, Transmission Electron Microscopy, and energy dispersive spectroscopy. Species of *Streptococcus oralis*, *Streptococcus mutans*, *Porphyromonas gingivalis*, Methicillin-sensitive *Staphylococcus aureus*, and Methicillin-resistant *Staphylococcus aureus* were used and tested using Minimum Inhibitory Concentration assays with concentrations of silver nanoparticles and simvastatin alone and in combination. Biofilm inhibition and viability tests were performed on titanium surfaces. Toxicity tests were also performed on *Galleria mellonella* moth larvae. Results: The silver nanoparticles had a spherical shape without the formation of aggregates as confirmed by Transmission Electron Microscopy. Dynamic Light Scattering revealed nanoparticles with an average diameter of 53.8 nm (±1.23 nm), a polydispersity index of 0.23 and a zeta potential of −25 mV (±2.19 mV). The silver nanoparticles inhibited the growth of the strains tested in the range of 0.001592 and 63.75, while simvastatin alone inhibited the growth of the same strains in the range of 3.125–62.5 µg/mL. The antibacterial activity test of the combination of the two substances showed a reduction in the Minimum Inhibitory Concentration of about two to eight times, showing synergistic effects on *Staphylococcus aureus* and additive effects on *Streptococcus oralis* and *Porphyromonas gingivalis*. As for biofilm, sub-inhibitory concentrations of the combination of substances showed better antibacterial activity in inhibiting the formation of *Streptococcus oralis* biofilm, and this combination also proved effective in eradicating already established biofilms compared to the substances alone. The combination of silver nanoparticles and simvastatin showed low toxicity to *Galleria mellonella* moth larvae. Conclusions: The results presented indicate that the combination of the two substances could be an alternative for the prevention and reduction of biofilms on implants. These findings open up new possibilities in the search for alternatives for the treatment of peri-implant infections, as well as the possibility of using lower doses compared to single drugs, achieving the same results and reducing potential toxic effects.

## 1. Introduction

Currently, the use of metal implants to replace missing teeth has become a customary practice, but despite the high success rate and long-term survival, patients may develop infections and inflammations in the peri-implant region, leading to implant failure [1,2,3].

Among the diseases that can affect the peri-implant region, the most common is mucositis, which is characterized by the involvement of only the surrounding soft tissues, followed by peri-implantitis, which is caused by progressive inflammation of the alveolar bone crest that supports the implant, resulting in bone loss and surgical failure [4,5,6,7].

Peri-implantitis sites have been associated with bacterial diversity with the growth of pathogenic anaerobic bacteria, specifically members of the “red complex” group, *Porphyromonas gingivalis*, *Tannerella forsythia*, *Treponema denticola*, and also other species from the *Treponema* I to III and *Synergisteteso* groups [8,9]. In addition, *Staphylococcus aureus*, *Pseudomonas aeruginosa*, and *Candida* sp. have been detected in cases of implant inflammation and have been associated with early implant failure [4,10,11,12].

Some of the individual’s pre-existing conditions, such as diabetes mellitus, smoking, and poor hygiene, may contribute to the development and progression of peri-implantitis [13]. However, the main etiology is the formation of biofilms, which are communities of microorganisms around implants that can trigger a host response resulting from the immunological interaction of toxins, antigens, and lipopolysaccharides with epithelial cells and granulocytes in the sulcus of the gingival mucosa [14].

It is important to emphasize that the different chemical composition of biomaterials used in implants is related to bacterial adhesion and succession during biofilm formation [15]. In particular, on titanium surfaces, the formation of an acquired film free of bacteria and rich in proteins such as proline, secretory IgA, α-amylase, and high molecular weight mucins provides an interface between the implant and initial colonizers such as *Streptococcus* sp. and *Actinomyces* sp. species that reach the film and titanium by fluid flow, Brownian motion, and chemotaxis [16].

*Streptococci* are highly prevalent during initial biofilm formation, and *Streptococcus oralis* in particular has been found to be an early colonizer of oral implants and associated with periodontitis [17]. Compared to other species such as *Acntinomyces naeslundi*, *Veillonela díspar* and *P. gingivalis*, it showed a higher potential for biomass formation [18]. Thus, the abundance of colonies of this species has been shown to favor the binding of pathogens to the biofilm through the expansion of signaling molecules and has been associated with greater tissue destruction during implant insertion [19,20].

The most common treatments are based on removal of the biofilm by mechanical debridement, which can be combined with chemical decontamination. However, the clinical outcomes of these treatments are lower than expected and there is no good evidence to support the usefulness of currently available chemical decontamination methods [21]. In addition, nonrational prophylaxis and treatment with antibiotics can cause adverse reactions and contribute to an increased risk of infection by resistant microorganisms such as *Enterobacter* sp., *Candida* sp., and *Staphylococcus* sp. [22,23,24,25]. In addition, inadequate doses may not reach poorly vascularized regions such as bone [25].

The development of new antibacterial compounds can be effective alternatives for both the control and prophylaxis of peri-implant infections. Among noble metal nanoparticles, silver nanoparticles (AgNP) have demonstrated biological activity and have therefore been used as highly effective, broad-spectrum agents for use in biomedical devices [26]. In addition, AgNP have exceptional physical properties, including high surface-to-volume ratio, high catalytic potential, and greater reactivity compared to their conventional form [27]. In dentistry, silver nanoparticles are used in the development of various antibacterial materials, such as orthodontic appliances, acrylic resins for prosthetics, composite resins for restorations, irrigation solutions, adhesives, and implant coatings [28].

The combination of AgNP with other antibiotics has shown a synergistic effect by increasing the antibacterial activity of already used antibiotics by redirecting them to specific targets and reducing antibacterial resistance [29,30].

AgNP are mainly synthesized by chemical and physical methods, which are generally more expensive and can be harmful to the environment due to the use of substances with specific biological risk potential [27]. Therefore, bio-based ecological approaches using bacteria, fungi, and plant extracts can be advantageous alternatives as they are economically viable, environmentally friendly, safe, and scalable [31]. Among the microorganisms used for the production of AgNP, fungi are considered true nanofactories due to the fact that they produce AgNP extracellularly and by biomimetic mineralization [32]. The production of silver nanoparticles by the fungus *Fusarium oxysporum* consists of the extracellular reduction of Ag^+^ ions to Ag^0^ in the presence of NADH-dependent reductases and electron-transporting quinones [31,32].

Another therapeutic approach that can be used to combat bacterial infections is the repurposing of drugs that have antibacterial activity as a side effect [33]. In this regard, statins are important lipid-lowering agents that reduce cholesterol synthesis by inhibiting the enzyme 3-hydroxy-3-methylglutaryl coenzyme A reductase-HMG-CoA, and have pleiotropic effects, i.e., effects that go beyond cholesterol reduction, such as antioxidant, anticarcinogenic, anticoagulant, anti-inflammatory, immunomodulatory, and antibacterial effects [34,35,36].

The antibacterial activity of isolated simvastatin has been demonstrated against *S. aureus* both in planktonic form and in biofilms [35] and in the inhibition of multi-species biofilms causing periodontal disease [37,38]. Similarly, AgNP have been tested for coating implants for antibacterial purposes [39,40,41].

The interaction between AgNP and simvastatin has previously been shown to be synergistic against standard strains of *S. aureus* [42], and the combination of these compounds showed antibacterial activity against biofilms of clinical strains of *S. aureus* and methicillin-resistant *S. aureus* (MRSA) [36]. Therefore, the aim of this study was to evaluate the antibacterial activity of the combination of simvastatin and AgNP on the formation and viability of mature biofilms on titanium discs against *Streptococcus oralis*.

## 2. Results and Discussion

### 2.1. Physical-Chemical Characterization of AgNP

**Figure 1:**
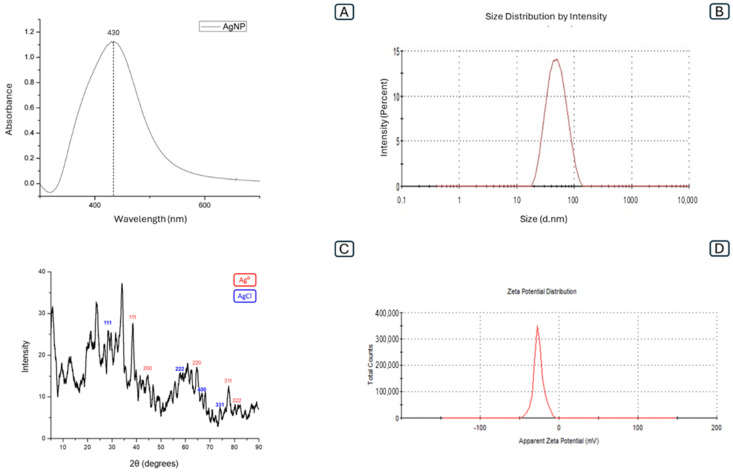
(**A**) UV-vis absorption spectrum of fungal filtrate after addition of AgNO_3_. (**B**) AgNP size distribution versus intensity (Z-average 53.8). (**C**) X-ray diffraction pattern of AgNP (**D**) Zeta potential distribution (−25.66 mV).

The synthesis of silver nanoparticles (AgNP) was carried out by the biosynthetic method using the fungus *Fusarium oxysporum*. The filtrate initially had a yellowish color, and after the addition of AgNO_3_, the solution turned yellowish-brown: the change in color of the solution is the first evidence of AgNP formation, was observed after 24 h and stabilized after 168 h, and was confirmed by UV-vis spectroscopy [43]. As shown in Figure 1A, there was a single peak with an absorbance value of 430 nm, which was attributed to the presence of surface plasmon resonance [44]. Thus, when metal nanoparticles are irradiated with light, the free electrons on their surfaces oscillate, causing some of the visible light to be absorbed, explaining the so-called plasmon resonance effect, which depends on the type of material, the size of the particle and its morphology [45,46]. The absorbance value found is in agreement with other studies using *Fusarium* sp. in AgNP biosynthesis, which found values between 415 and 440 nm [32,47,48].

The UV-vis analysis of the dispersion of AgNP was evaluated at pH 7.4, a temperature of 28 °C, and a fungal biomass concentration of 10%, using a concentration of 3 mM AgNO_3_. The entire biosynthetic process was carried out in the dark.

As shown in Figure 1C, the evaluation of the crystalline nature of AgNP by X-ray diffraction (XRD) analysis showed Bragg reflections at 2ϴ = 38.28°, 44.53°, 64.55°, 77.64° and 81.33°, which can be indexed as diffraction planes (111), (200), (220), (311) and (222), confirming the presence of AgNP with face-centered cubic crystalline structure [49]. Although to a lesser extent, other diffraction peaks were observed at 2ϴ = 27.9°, 57.6°, 67.6°, 74.6°, representing facets of silver chloride nanoparticles (Ag/AgCl), a compound most commonly found in the final product of AgNP synthesized by biological routes [50], so they could be related to the partial oxidation of AgNP during synthesis due to the presence of chloride ions from the culture medium or metabolites from the fungal biomass. [51,52]. In addition, the AgNP produced showed a reflection at 2ϴ = 33.9, which refers to the plane (111) of Ag_2_O. It is believed that the presence of oxide in the sample is due to the fact that the AgNP were not synthesized in an inert atmosphere [53].

Similar XRD patterns have been reported in studies using nanoparticles synthesized by *F. oxysporium* and in biogenic laccase nanoparticles produced by *Trametes versicolor* [54,55,56].

**Figure 2:**
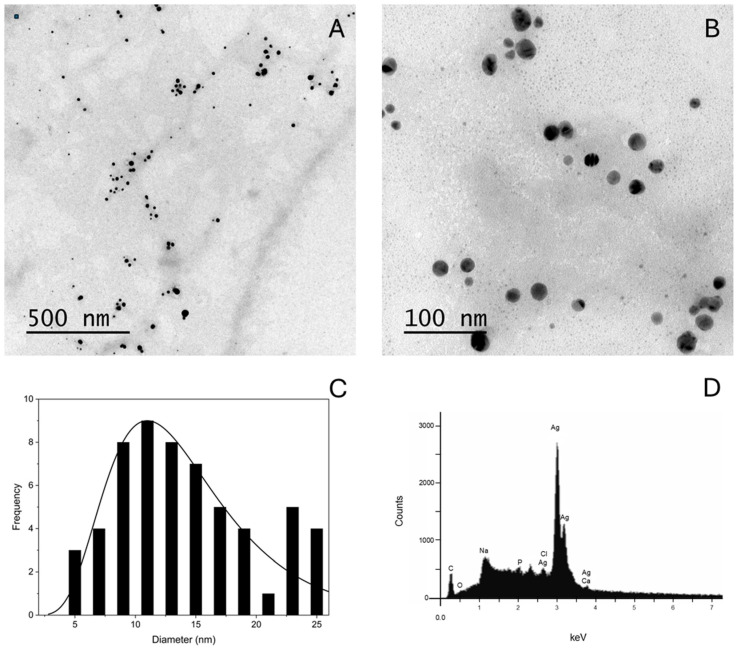
(**A**) Micrographs of biosynthesized AgNP obtained by TEM on scale of 500 nm. (**B**) AgNP on a scale of 100 nm. (**C**) Histogram of the size distribution of AgNP. (**D**) Energy dispersive X-ray (EDX) spectrum of AgNP.

The EDS spectrum is an analytical technique used to determine the elemental composition and quantify specific elements [57]. The analyses presented in Figure 2D show a strong silver signal at 3 kev of oxygen ~0.5 kev, chlorine ~2.7 kev and carbon ~0.3 kev. In addition, the weight percentages of silver were 82.74%, oxygen 2.67%, chlorine 0.20% and carbon 12.37%, as shown in Table 1, confirming that the AgNP were the main products of biogenic synthesis. The presence of carbon as the second most contributing element may come from the proteins surrounding the nanoparticles. The average diameter of the nanoparticles was 53.8 nm (±1.23), as shown in Figure 1B, with 0.23 of polydispersity index (PDI), which indicates that the AgNP were monodisperse with a narrow size distribution [58]. In addition, the zeta potential was −25.66 ± (2.19 mV), a high zeta potential value, above ±30 mV, confers stability in colloidal systems so that the dispersion may resist aggregation, as shown in Figure 1D [59]. The spherical shape of the nanoparticles was confirmed by Transmission Electron Microscopy (TEM), and homogeneous and dispersed particles without aggregates were observed, as shown in Figure 2A,B.

### 2.2. Minimum Inhibitory Concentration (MIC) Assay and Association of AgNP and SIM

AgNP showed MIC values between 0.0016 to 63.7 µg/mL against all tested strains. In addition, simvastatin presented MIC values between 3.125 and 62.5 μg/mL. Lower MIC values, such as those presented by AgNP, indicated better antibacterial efficacy on *P. gingivalis*, *S. mutans* and *S. oralis* species. All microorganisms were susceptible to the standard antibacterials, vancomycin (VAN), tested in a concentration range between 6.25 and 0.0031 μg/mL, ampicillin (AMP), 1.55 and 0.00076 μg/mL, and metronidazole (METRO), 3.12 and 0.0015 μg/mL, as shown in Table 2.

These results are corroborated by studies evaluating the antibacterial activity of simvastatin against *S. aureus* strains, including methicillin-resistant *Staphylococcus aureus* (MRSA), which found MICs similar to the results of the present study [35,36]. They are also corroborated by studies that evaluated the effect of simvastatin alone and in combination with antibiotics on microorganisms present in oral infectious processes [38,56]. Finally, isolated AgNP concentrations that inhibited the growth of microbial strains were found in other studies evaluating AgNP against *S. aureus* and MRSA strains [36] and oral pathogens [60,61].

AgNP combined with simvastatin showed synergism in *S. aureus* ATCC 29213 but were not effective against other strains of *S. aureus* and *S. mutans*. For *S. oralis* and *P. gingivalis*, there was an additive effect with AgNP–SIM, as shown in Table 3. For *P. gingivalis*, *S. oralis* and *S. aureus*, the combination of SIM and AgNP reduced the MIC of the compounds by approximately two to eight times compared to the isolated compounds.

These results can be compared to previous studies that showed synergistic antibacterial activity of AgNP with tetracycline in reducing the inhibition range in *P. aeruginosa*, *E. coli* and *K. pneumoniae* [62], the combination of AgNP with amoxicillin in restorative material showed superior antibacterial effect compared to the isolated substances in *S. aureus* and *S. mutans* [63].

Other substances have shown synergistic effects in combination with AgNP, such as the interaction with tyrosol, an antibacterial molecule secreted by *Candida albicans*. The interaction of this molecule with AgNP inhibited the growth of *C. albicans* and *S. mutans* [64]. Another study evaluated the interaction between AgNP and oregano essential oil, and demonstrated a synergistic or additive effect against gram-positive and gram-negative bacterial species [65]. The interaction of AgNP and curcumin demonstrated a synergistic effect in *P. aeruginosa* and *E. coli* [66], and finally, the interaction between AgNP and simvastatin was effective against Methicillin-sensitive *Staphylococcus aureus* (MSSA), Methicillin-resistant *Staphylococcus aureus* (MRSA), and multidrug-resistant *Escherichia coli* [36,42].

### 2.3. Inhibition of Biofilm Formation and Viability Assay of S. oralis

**Figure 3:**
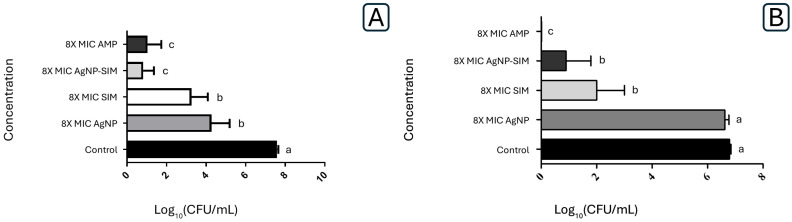
(**A**) *S. oralis* adhesion inhibition assay under exposure to AgNP, SIM, AgNP–SIM and AMP. (**B**) *S. oralis* biofilm viability assay under exposure to AgNP, SIM, AgNP–SIM and AMP for 24 h. The amount of biofilm formed is represented by means and standard deviations of log CFU/mL (*p* < 0.05, ANOVA, Tukey’s post-test). Letters indicate statistical differences between the experimental groups compared to the control group, represent by the letter a.

Since the AgNP–SIM combination had positive effects against *S. oralis* 10,557 and due to the ability of this species to form biofilms [67], they were used for inhibition and viability tests in biofilm on titanium discs. Both simvastatin and AgNP, when isolated, similarly inhibited the formation of *S. oralis* biofilm. However, the combination of AgNP–SIM had a greater effect on inhibiting biofilm formation, which could be compared to ampicillin, as shown in Figure 3) (*p* > 0.05, ANOVA, Tukey’s post-test).

**Figure 4:**
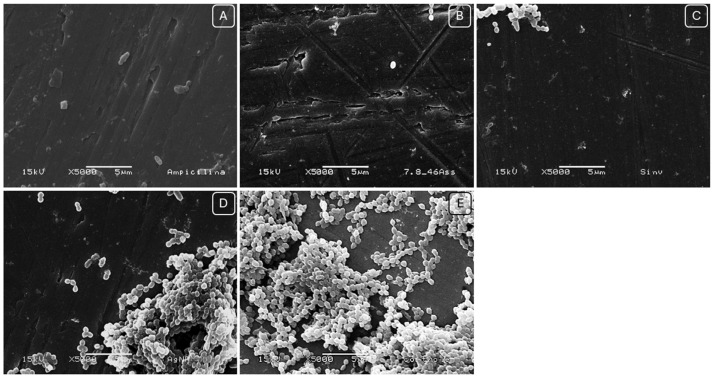
*S. oralis* biofilm on titanium disk treated with: (**A**) 8 × MIC of the combination of AgNP and SIM. (**B**) 8 × MIC of SIM. (**C**) 8 × MIC of AgNP. (**D**) 8 × MIC of Ampicillin. (**E**) No treatment, after 48 h of biofilm formation.

Regarding the reduction of biofilm formed on titanium discs, AMP showed the best result, followed by the combination of AgNP–SIM and SIM alone. However, the discs treated with AgNP alone did not show any reduction in the number of viable cells. As observed in Figure 4. Scanning electron microscope (SEM) images confirmed these results, with a significant reduction in biofilm mass for the combination of AMP and AgNP–SIM, but no reduction for AgNP–SIM.

A study using AgNP between 10 and 20 nm coated with citrate enhanced the effect of tobramycin against biofilms of clinical strains of *P. aeruginosa* [30]. Another study testing the interaction of AgNP and chlorhexidine reported greater efficacy against multi-species biofilms compared to the isolated substances [48]. Finally, a previous study from our research group showed a synergistic effect of AgNP and simvastatin against a standard strain of *S. aureus* biofilm [36].

Due to its pleiotropic effects, simvastatin has demonstrated antibacterial activity against opportunistic bacteria and oral pathogens [35,36,38,42,68]. Previous studies have shown that simvastatin inhibits *S. aureus* biofilm at a concentration of 4 × MIC (62.5 µg/mL) and affects the production of insoluble extracellular polysaccharide [54]. Another study demonstrated that simvastatin inhibits *P. gingivalis* biofilm, which is commonly found in peri-implantitis [8], and a 79% reduction in metabolic activity was observed in multispecies biofilm of periodontal pathogens [38].

Similarly, AgNP reduced biofilm formation and can be compared with previous studies that showed an inhibitory effect of AgNP on oral biofilms clinically isolated from patients with and without periodontal disease [69], as well as a formulation containing AgNP that prevented biofilm formation of several species of *Streptococcus* and *E. faecalis* in vitro [70]. In addition, AgNP inhibited the formation of multidrug-resistant biofilms of *P. aeruginosa* [71].

Isolated AgNP had no effect on consolidated biofilms, but not because they developed resistance. Compared to other antibacterials, AgNP has multiple antibacterial mechanisms, including reduced ATP production by Ag^+^ ions, generation of reactive oxygen species, and damage to the DNA and bacterial cell membrane [28,72]. Particularly against biofilms, AgNP interact with lipids and lipopolysaccharides (LPS), causing structural disruption. The penetration of nanoparticles into the biofilm depends on many factors, such as biofilm maturity, composition and chemistry of its surface, nanoparticle size, concentration, charge and surface chemistry [73].

Titanium discs were in contact with AgNP treatment for 48 h, which may not be sufficient time for biofilm eradication. Previous studies have shown a reduced antibacterial capacity of AgNP used as reticular canal irrigators, which was attributed to the short period of interaction as well as the low concentration (94 ppm) [74]. Another study demonstrated a potential effect of AgNP in the eradication of biofilm formed in root dentin when used as a treatment for 7 days, but the effect was limited as an irrigating solution, showing that the bactericidal treatment of biofilm is dependent on a longer period of exposure [75].

Another possible reason may be related to the possible interaction of these nanoparticles with the stabilizing proteins from the fungal filtrate. It has been reported that prior washing of AgNP resulted in improved antibacterial activity and was associated with a lower concentration of these proteins [76]. It is important to remember that the antibacterial activity of AgNP depends on the complexes they form with proteins present in biofluids that are responsible for coating the nanoparticle, the so-called protein corona, which determines the physical, chemical and biological properties of the nanoparticles [77,78]. These complexes can interfere with antibacterial activity, so it is the proteins in the corona that interact with cells, not the nanoparticles themselves [79,80].

Gnanadhas et al. [81] observed that unencapsulated AgNP showed no antibacterial activity in the presence of serum proteins due to interaction with bovine serum albumin (BSA). However, AgNP encapsulated with citrate or polyvinylpyrrolidone exhibited antibacterial properties due to minimized interactions with serum proteins. In the present study, the titanium discs were soaked in human saliva to form the acquired pellicle, which allowed bacterial adhesion. In addition, the culture medium for biofilm growth contained mucin, a glycoprotein present in saliva. Thus, proteins present in these substrates could interact with AgNP. However, these interactions need to be further investigated.

### 2.4. Simvastatin and AgNP Toxicity In Vivo

**Figure 5:**
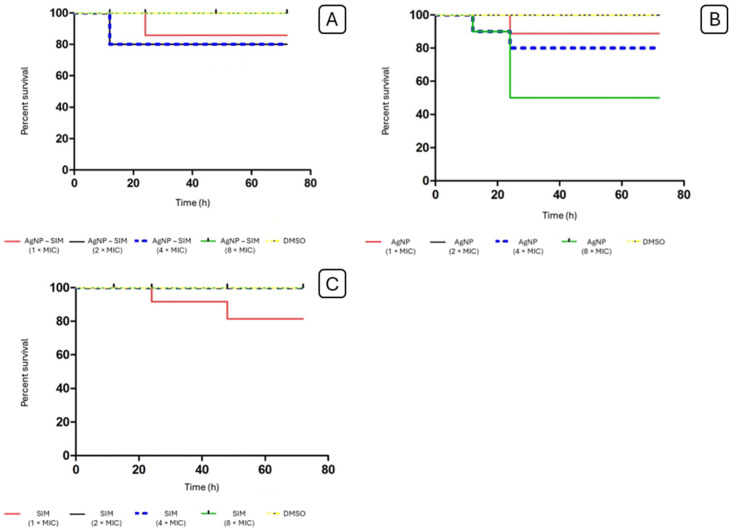
Survival curve of *G. mellonella* after treatment with different concentrations of (**A**) AgNP–SIM, (**B**) AgNP, and (**C**) SIM.

After 12 h exposure to 2 × MIC and 4 × MIC concentrations of the AgNP–SIM combination, the viability of *G. mellonella* larvae was reduced to 80%. After 24 h of evaluation, the 1 × MIC concentration of AgNP–SIM reduced viability to 90%. There was no reduction in viability at the 8 × MIC concentration or in the DMSO group used as a control. The AgNP group showed a reduction in larval viability to 90% at the 4 × MIC and 8 × MIC concentrations after 12 h exposure, and a reduction to 80% and 60% at these concentrations after 24 h exposure, *p* = 0.029. The simvastatin group showed a reduction in larval viability to 90% at the MIC concentration after 24 h and a reduction to 80% at the MIC concentration after 48 h (*p* = 0.091). Table 4 shows the doses of each compound injected into *G. mellonella*, as shown in Figure 5.

**Figure 6:**
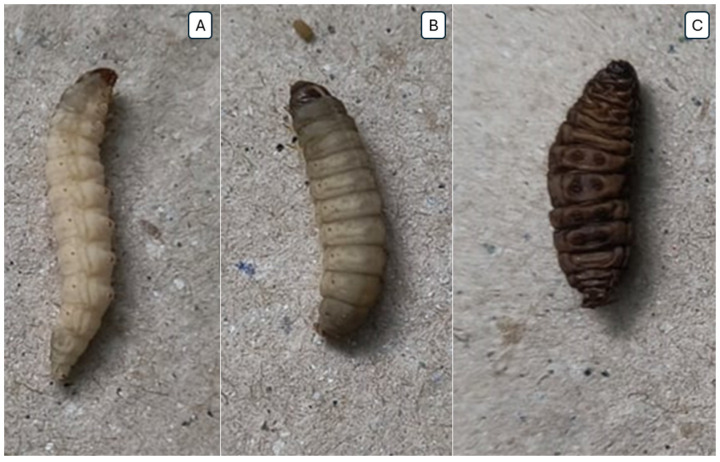
*Galleria mellonella* larvae. (**A**) Healthy larva without treatment; (**B**) Partially melanized larvae not counted as alive; (**C**) Melanized larvae not counted as alive.

The toxicity assay of the AgNP–SIM combination was performed on *Galleria mellonella*, as shown in Figure 6, a model that replaces the use of mammals due to the similarity of its immune system, in addition to being widely accepted in the scientific literature [82]. The use of this model has been described in the activity of biogenic AgNP in larvae infected with *P. aeruginosa* [83]. It also has been tested for screening potential drugs against *Staphylococcus* strains, such as daptomycin and vancomycin [84]. At the highest concentrations (8 × MIC) of AgNP–SIM to test the biofilm viability, the larvae did not reduce the viability, however, at lower concentrations, viability was reduced by up to 80%, which can be explained by the higher levels of DMSO solvent used to solubilize SIM. AgNP showed greater toxicity only at the highest concentration (8 × MIC), where viability was reduced to 50%. In a similar study, the doses of isolated AgNP tested did not show a reduction in larval viability over the periods evaluated, but the highest dose tested was 5 mg/kg [85]. Since the concentrations of each substance are lower in the AgNP–SIM combination, this may explain the reduced toxicity of this combination. Thus, we can conclude that the AgNP–SIM combination is less toxic than AgNP alone in terms of MIC values. Our results showed that the AgNP–SIM combination may be an alternative for controlling infection on implants, which provides opportunities for further testing in preclinical studies.

## 3. Materials and Methods

### 3.1. Chemicals and Experimental Groups

Simvastatin (SIM), donated by EMS Pharma (Hortolândia, Sao Paulo, Brazil). Amoxicillin (AMOX, Sigma-Aldrich, St. Louis, MO, USA), metronidazole (METRO, Sigma-Aldrich, St. Louis, MO, USA), vancomycin (VAN, TEUTO Anápolis, Brazil) and ampicillin (AMP, Sigma-Aldrich, St. Louis, MO, USA) were used as standard antibacterials. SIM, and METRO were diluted in dimethyl sulfoxide (DMSO) to final concentrations of 2–2.5%, while AMOX, VAN, and AMP were dissolved in sterile distilled water.

The formulations were distributed in microplate wells as follows: (a) experimental groups (culture medium + bacteria + statin or antibacterial standard); (b) positive control (culture medium + bacteria); (c) vehicle control (culture medium + bacteria + DMSO); (d) negative control (culture medium + statin or standard antibacterial); (e) negative control of the medium (culture medium). All tests were performed with six replicates on at least two separate occasions.

### 3.2. Biosynthesis of Silver Nanoparticles

Silver nanoparticles (AgNP) were biosynthesized by fungi, according to the method previously described [32]. The strain 551 of the fungus *Fusarum oxysporum* used was obtained from the culture collection of molecular genetics laboratory of ESALQ-USP, Piracicaba-SP, Brazil. The fungus was grown on malt agar (Difco^®^) containing 0.5% yeast extract, 2% malt extract, 2% agar and distilled water for 7 days at 28 °C. Then, 10 g of fungal biomass (previously washed) from the culture medium were added to 100 mL of sterile distilled water and incubated for 72 h at 28 °C. After that, the supernatant was separated from the fungal biomass by vacuum filtration and AgNO_3_ (Sigma-Aldrich^®^) was added for a final concentration of 3 mM. The system solution was incubated at 28 °C in the absence of light until AgNP were formed. The observation of AgNP formation was performed visually and by colorimetric analysis using a spectrophotometer (UV-vis) until the formation of nanoparticles at 430 nm. After purification, the AgNP were characterized on filters with 0.22 mm membranes (Orion Cientific, Rio de Janeiro, Brazil).

### 3.3. Characterization of Silver Nanoparticles

The hydrodynamic size, polydispersity index (PDI) and zeta potential of AgNP were determined by Dynamic Light Scattering—DLS (ZetaSizer NanoZS—Malvern Panalytical, Malvern, UK). Transmission Electron Microscopy—TEM (Jeol JEM-1400, Peabody, MA, USA) was performed to confirm AgNP morphology and size. X-ray diffraction (XRD) spectroscopy technique, XDR 7000 (Shimadzu, Kyoto, Japan) was used to determine and confirm the crystalline structure of AgNP and energy dispersive X-ray spectroscopy (EDS) (Vantage V.1.4 Rev. B, Noran Instruments, Middleton, WI, USA) was used to detect the elements on the surface of AgNP.

### 3.4. Bacterial Strains and Cultivation Conditions

Strains of *S. aureus* ATCC 29213 (MSSA), *S. aureus* ATCC 6538, *S. aureus* ATCC 43300 (MRSA; mecA gene present), *S. aureus* MRSA 33591, *Streptococcus oralis* ATCC 10557, *Streptococcus mutans* UA 159, and *Porphyromonas gingivalis* W83 and ATCC 33277 were used.

Stock cultures of *S. aureus* were grown in Mueller Hinton Broth (MHB, Difco Co., Detroit, MI, USA) medium for 24 h under aerobic conditions in an incubator (Solab, Piracicaba, Sao Paulo, Brazil) at 37 °C. Facultative anaerobic microorganisms *S. mutans* and *S. oralis* were cultured in Brain Heart Infusion Broth, (BHI, Difco Co., Detroit, MI, USA) medium for 24 h in an incubator (Sanyo Electric Co., Osaka, Japan, MCO-19AIC) at 37 °C with 5% CO_2_ and *Porphyromonas gingivalis* were cultured in Tryptic Soy Agar (TSA, Difco Co, Detroit, MI, USA), supplemented with 7% sheep blood, 0.2% Yeast Extract (YE—Difco Co., Detroit, MI, USA), 5 μg/mL Hemin (Sigma Aldrich.—St. Louis, MO, USA, H5533), and 1 μg/mL Menadione (Sigma Aldrich.—St. Louis, MO, USA, M5625), incubated under anaerobic conditions for 48 h (80% N_2_, 10% CO_2_, 10% H_2_ (MiniMacs Anaerobic Workstation; Don Whitley Scientific, Shipley, UK) at 37 °C. The cultures were stored in Tryptic Soy Broth (TSB, Difco Co., Detroit, MI, USA) with 20% glycerol, at −80 °C.

The bacterial inoculum was adjusted for MIC and Checkerboard Microdilution Assay for an optical density of 0.08–0.1 (660 nm) grown in MHB in aerobiosis for 24 h for *S. aureus*. For the assays with *P. gingivalis*, an optical density of 0.5 (660 nm) was used, and after that, a 10-fold dilution was performed in 1.55% Tryptic Soy Broth added with 1.48% BHI, 0.2% YE, 5 μg/mL Hemin and 1 μg/mL Menadione, [86]. For *streptococcal* inoculum, cultures were grown for 24 h and suspensions were adjusted to an absorbance range of 0.08–0.1 (625 nm) in MHB [86].

### 3.5. Minimum Inhibitory Concentration (MIC) Assay

The minimum inhibitory concentration (MIC) was determined by microdilution assay in culture medium in 96-well microplates according to the guidance of the Clinical and Laboratory Standards Institute—CLSI [86]. Briefly, different concentrations of simvastatin (250 to 0.12 µg/mL) and AgNP (127.5 to 0.06 µg/mL) and standard antibiotics (10–0.001 μg/mL) were added by 2-fold dilution. Then, 100 μL of bacterial suspension prepared according to item 3.4 were inoculated and the 96-well plates were incubated under aerobic conditions for 24 h (*S. aureus*), under anaerobic conditions for 48 h (*P. gingivalis*) or in 5% CO_2_ for 24 h (*streptococci*). The bacterial suspensions were adjusted according to the turbidity equivalent to the Mc Farland standard, resulting in a concentration of 1 × 10^8^ CFU/mL. Then, 100 µL of the suspensions were diluted in 9.9 mL of MHB to obtain a suspension of 1 × 10^6^ CFU/mL. Finally, 100 µL of the suspension was added to the plate obtaining a final concentration of 1 × 10^5^ CFU/mL. The lowest concentration with any visible bacterial growth was taken as MIC. In addition, bacterial growth was assessed by optical density measurement and through the addition of 30 µL resazurin solution 0.01% [87]. This experiment was performed in triplicate of at least two independent assays.

### 3.6. Antibacterial Combination Assay (Checkerboard Assay)

The Checkerboard Microdilution Assay was used to verify the synergistic activity between simvastatin and AgNP, combining different concentrations of the compounds as previously described [35,88]. Different concentrations of SIM and AgNP combinations were diluted in MHB medium in 96-well plates. The concentrations of each substance used were the same as the MIC. After that, a 100 µL aliquot of bacterial suspension was added, at a final concentration of 1 × 10^5^ CFU/mL. The plates were incubated according to the culture conditions of each strain. The plates were visually analyzed for turbidity and the absorbance was evaluated in a spectrophotometer (θ = 660 nm). Finally, 30 μL of 0.01% resazurin dye was added to each well, which were incubated for two hours and read.

To qualify the interaction between the two compounds, the fractional inhibitory concentration index (FICI) was calculated using combined MIC of both compounds (CIMAB) and MIC of each compound alone (CIMA), using the following equation:

Σ = FICIA + FICIB = MICAB/MICA + MICBA/MICB.

The FICI was interpreted according to the following index: ≤0.5, synergistic interaction effect; >0.5 and ≤1.0, additive interaction effect; >1 and <4, indifferent; and ≥4, antagonistic interaction effect [89].

### 3.7. Inhibition Assay of S. oralis Adhesion on Titanium Discs

Biofilm assays were performed in 24-well polystyrene plates coupled with sterilized titanium discs measuring 4.2 mm thick by 8 mm in diameter—Porous ^®^ surface (Conexão prosthesis systems, Arujá, Sao Paulo, Brazil) secured by a metal apparatus. Because we used human saliva from donors, this study was previously approved by the research ethics committee (CEP) #2.805.995.

#### 3.7.1. Saliva Preparation

Saliva was obtained from healthy volunteers in 10 mL aliquots. The sample was treated with 2.5 mmol L−1 of phenylmethylsulfonyl fluoride (Sigma Aldrich, St. Louis, MO, USA) to reduce salivary protein aggregation. After treatment, saliva was centrifuged at 10 min, 4 °C, 3800× *g* and the supernatant obtained was diluted (1:1) with AB solution (KCl—0.93 g, CaCl_2_—0.02 g, KHPO_4_—0.034 g, MgCl_2_—0.005 g). The sample was then filtered and sterilized using a 0.22 μm Millex GV filter (Millipore, Millipore Corporation, Bedford, MA, USA). The saliva suspension was added to a 24-well plate and the titanium discs were positioned vertically, held by a metal wire, and then incubated in an orbital shaker (211DS Shaking Incubator, Labnet International, Inc., Edison, NJ, USA) at 37 °C, 60 rpm, for 2 h, for the formation of saliva biofilm.

#### 3.7.2. Inhibition of Biofilm Formation and Biofilm Viability Assay of *S. oralis*

For biofilm formation, cultures of *S. oralis* ATCC 10,557 were grown in BHI medium supplemented with 2.5 g/L mucin, 1.0 g/L yeast extract, 0.1 g/L cysteine, 2.0 g/L sodium bicarbonate, 5.0 mg/mL hemin, and 1.0 mg/mL menadione [90].

After coating the discs with saliva film, they were placed in 24-well plates in a vertical position. For the biofilm formation inhibition assay, the treatments were added at time 0 h, before biofilm formation. In each well, 3 mL of culture medium with 8 × MIC concentrations of SIM, AgNP, AgNP–SIM and 0.3 mL of *S. oralis* culture at 1.0 × 10^8^ CFU/mL were added to the wells containing the titanium discs. The plates were incubated for 48 h, in 5% of CO_2_, at 37 °C. After this period, the discs were removed from the wells and washed in sterile saline solution to remove non-adhered cells and placed in tubes containing 5 mL of sterile 0.9% NaCl. The tubes were vortexed for one minute, after which they were sonicated at 5% amplitude, 6 pulses of 9.9 s with 5 s intervals for another minute (Vibra Cell 400 W, Sonics & Materials Inc., Newtown, CT, USA). The bacterial suspension was then diluted (10–100,000 times) and 0.01 mL of each dilution was plated on BHI agar medium. The plates were incubated in 5% CO_2_ at 37 °C for 48 h. After this period, the colonies were quantified and the colony forming units per mL (CFU/mL) were calculated.

For the mature biofilm viability test, concentrations of each compound were added 24 h after biofilm formation. After adding the treatments, the discs were incubated for an additional 24 h in 5% CO_2_ at 37 °C. After 48 h of contact with the substances, the discs were removed from the wells, washed with 0.9% NaCl solution and placed in tubes containing 5 mL of sterile 0.9% NaCl. The tubes were vortexed for one minute and then sonicated as previously described. Finally, the bacterial suspension was diluted and plated on BHI agar medium, and the plates were incubated for 48 h. After this period, the CFU/mL of each sample was calculated.

#### 3.7.3. Scanning Electron Microscopy (SEM)

Scanning Electron Microscopy analyses were performed on *S. oralis* biofilms formed for 24 h and treated for 48 h with 8 × MIC concentrations of SIM, AgNP, AgNP–SIM. After growth and treatment of the biofilms on titanium discs, they were washed in 0.9% NaCl solution and after washing, the discs were fixed in 10% glutaraldehyde solution in Phosphate buffered saline solution (PBS) for 30 min. Then, the biofilms were immersed in 90% and 99% ethanol solutions for one minute, respectively. Finally, the discs were dried at room temperature for 12 h and the samples were fixed on a brass stub, and then metallized with gold. The discs were then evaluated in a Scanning Electron Microscope (JEOL, JSM5600LV, Tokyo, Japan) and images were taken at 15 KV.

#### 3.7.4. Acute Toxicity Assay In Vivo in the *Galleria mellonella* Model

This assay was performed to evaluate possible acute toxic effects of the combination of AgNP–SIM, SIM and isolated AgNP. A total of 10 larvae weighing between 0.2 and 0.3 g were selected for each group. The larvae remained in the refrigerator for 30 min to facilitate the administration of the treatment solutions: 2.5% DMSO solution (diluent control) and 1 × MIC AgNP–SIM, 2 × MIC AgNP–SIM, 4 × MIC AgNP–SIM and 8 × MIC AgNP–SIM, 1 × MIC of SIM, 2 × MIC of SIM, 4 × MIC of SIM and 8 × MIC of SIM and 1 × MIC of AgNP, 2 × MIC of AgNP, 4 × MIC of AgNP and 8 × MIC of AgNP. The substances were injected into the hemocoel of each larva at the last progeny left using a 25 µL Hamilton syringe (Hamilton, Reno, NV, USA). The larvae were incubated at 30 °C and their survival was recorded at 12 h, 24 h, 48 h and 72 h intervals. Larvae that did not move after touching and showed myelination were counted as dead [91].

### 3.8. Statistics

The data were compared with the control group by analysis of variance (ANOVA) and Tukey’s post-test. The analyses were performed with Bioestat 5.0 (Mamirauá, Belém, Brazil) and GraphPad Prism 8.0 (San Diego, CA, USA). For all comparisons, a “*p*” value of <0.05 was selected as the criterion for statistical significance. Survival assays were analyzed using Kaplan–Meier curves and Mantel–Cox tests.

## 4. Conclusions

Here, AgNP were biologically produced and their association with simvastatin showed an additive and synergistic effect against species of oral biofilm. Also, this association was able to inhibit and reduce the protection of the *S. oralis* biofilm in the titanium discs, with low in vivo toxicity tested in *Galleria mellonella*.

The association could be used as a potential drug in titanium implant coatings, contributing to the success of osteointegration. In this way, it would be guaranteed that concentrations close to or even above the MIC would not reach local infection or prevent microbial colonization. In addition, to date, there are no reports of antibacterial resistance to these drugs, which overcome the use of standard antibacterials. The synergistic effects provide a reduction in the concentration of both, which could reduce possible toxic effects.

## Figures and Tables

**Table 1:** List of elements and their composition obtained in EDX analysis of AgNP.

Element	Weigth %	Atomic %
C	12.37	50.78
O	2.67	8.22
P	1.00	1.59
Cl	0.60	0.84
Ag	82.74	37.81
Ca	0.61	0.75

**Table 2:** Simvastatin MIC, AgNP and standard antibiotics: vancomycin (VAN), ampicillin (AMP), and metronidazole (METRO).

Microorganisms	MIC Simvastatin (µg/mL)	CIM—AgNP (µg/mL)	MIC—Antibiotics (µg/mL)
*S. aureus* MRSA 43300	62.5	63.75	VAN 1.56
*S. aureus* MRSA 33591	62.5	31.88	VAN 1.56
*S. aureus* ATCC 29213	31.25	31.88	VAN 1.56
*S. aureus* ATCC 6538	31.25	31.88	VAN 1.56
*S. oralis* ATCC 10557	15.62	15.94	AMP 0.024
*S. mutans* UA159	31.25	15.94	AMP 0.048
*P. gingivalis* ATCC W83	6.25	0.001592	METRO 0.195
*P. gingivalis* ATCC 33277	6.25	0.001592	METRO 0.390

**Table 3:** Comparison of simvastatin MIC, AgNP, standard antibiotics alone or in combination, FICI values and pharmacological interaction of the drug combination against oral bacteria.

Microorganisms	MIC SIM (µg/mL)	MIC AgNP (µg/mL)	MIC Antibiotic (µg/mL)	MIC SIM/AgNP (µ/mL)	FICI	Interaction
*S. aureus* MRSA 43300	62.5	63.75	VAN 1.56	62.5–63.75	2	----
*S. aureus* MRSA 33591	62.5	31.88	VAN 1.56	62.5–31.88	2	----
*S. aureus* ATCC 6538	31.25	31.88	VAN 1.56	31.25–31.88	2	----
*S. aureus* ATCC 29213	31.25	31.88	VAN 1.56	7.8–15.94	0.32	Synergistic
*S. mutans* UA159	31.25	15.94	AMP 0.048	31.25–15.94	2	----
*S. oralis* ATCC 10557	15.62	15.94	AMP 0.024	7.81–7.97	0.73	Additive
*P. gingivalis* ATCC W83	6.25	0.001592	METRO 0.195	0.781–0.00079	0.75	Additive
*P. gingivalis* ATCC 33277	6.25	0.001592	METRO 0.390	3.125–0.00079	1	Additive

**Table 4:** Doses of compounds injected into *G. mellonella*.

Acute Toxicity Assessment—*G. mellonella*				
Compound (mg/Kg)	1 × MIC	2 × MIC	4 × MIC	8 × MIC
AgNP—SIM	26–53.1	52–103.3	104–212.5	208–423
AgNP	106.3	212.5	426	850
SIM	104.2	208.4	416.7	833

## Data Availability

Data are contained within the article.
